# The SPARKLE registry: protocol for an international prospective cohort study in patients with alpha-mannosidosis

**DOI:** 10.1186/s13023-020-01549-8

**Published:** 2020-09-29

**Authors:** Julia B. Hennermann, Nathalie Guffon, Federica Cattaneo, Ferdinando Ceravolo, Line Borgwardt, Allan M. Lund, Mercedes Gil-Campos, Anna Tylki-Szymanska, Nicole M. Muschol

**Affiliations:** 1 University Medical Center Mainz, Langenbeckstr. 1, 55131 Mainz, Germany; 2 Hôpital Femme Mère Enfant, Lyon, France; 3 Chiesi Farmaceutici S.p.A., Parma, Italy; 4 Copenhagen University Hospital, Rigshospitalet, Copenhagen, Denmark; 5 Reina Sofía University Hospital, IMIBIC, CIBEROBN, Córdoba, Spain; 6 The Children’s Memorial Health Institute, Warsaw, Poland; 7 International Center for Lysosomal Disorders (ICLD), University Medical Center Hamburg-Eppendorf, Hamburg, Germany

**Keywords:** Alpha-mannosidosis, Recombinant alpha-mannosidase, Velmanase alfa, Patient registry, Enzyme-replacement therapy

## Abstract

**Background:**

Alpha-mannosidosis is a lysosomal storage disorder caused by reduced enzymatic activity of alpha-mannosidase. SPARKLE is an alpha-mannosidosis registry intended to obtain long-term safety and effectiveness data on the use of velmanase alfa during routine clinical care in patients with alpha-mannosidosis. It is a post-approval commitment to European marketing authorization for Velmanase alfa (Lamzede^®^), the first enzyme replacement therapy for the treatment of non-neurologic manifestations in patients with mild to moderate alpha-mannosidosis. In addition, SPARKLE will expand the current understanding of alpha-mannosidosis by collecting data on the clinical manifestations, progression, and natural history of the disease in treated and untreated patients, respectively.

**Results:**

The SPARKLE registry is designed as a multicenter, multinational, noninterventional, prospective cohort study of patients with alpha-mannosidosis, starting patient enrollment in 2020. Patients will be followed for up to 15 years. Safety and effectiveness as post-authorization outcomes under routine clinical care in patients with treatment will be evaluated. The primary safety outcomes are the rate of adverse events (anti-velmanase alfa-immunoglobulin G antibody development, infusion-related reactions, and hypersensitivity). Secondary safety outcomes include the evaluation of medical events, change in vital signs, laboratory tests, physical examination, and electrocardiogram results. The primary effectiveness outcome is a global treatment response rate, evaluated as the individual aggregate of single endpoints from pharmacodynamic, functional, and quality-of-life effectiveness outcomes; secondary effectiveness outcomes are to characterize the population of patients with alpha-mannosidosis with regard to clinical manifestation, progression, and natural history of the disease. Any patient in the European Union with a diagnosis of alpha-mannosidosis who is willing to participate will likely be eligible for inclusion in the registry. Publications to disseminate scientific insights from the registry are planned.

**Conclusion:**

This study will provide real-world data on the long-term safety and effectiveness of velmanase alfa in patients with alpha-mannosidosis during routine clinical care and increase the understanding of the natural course, clinical manifestations, and progression of this ultra-rare disease.

## Background

Alpha-mannosidosis (OMIM 248500) is an ultra-rare monogenic disorder caused by alpha-mannosidase deficiency (Enzyme Commission number: 3.2.1.24) and has an estimated worldwide prevalence of 1:500,000–1:1,000,000 live births [1]. However, as alpha-mannosidosis is an underdiagnosed disease, the real prevalence may be higher [2]. Alpha-mannosidosis is an autosomal recessive, lysosomal-storage disease resulting from mutations in the *MAN2B1* gene, located on chromosome 19 (19 p13.2-q12) [3]. As alpha-mannosidase is a lysosomal enzyme involved in glycoprotein catabolism, reduced activity results in impaired degradation of glycoproteins and leads to an accumulation of mannose-rich oligosaccharides in various tissues [4].

Intracellular accumulation of mannose-rich oligosaccharides leads to different clinical symptoms, including hearing impairment, intellectual disabilities, impairment of motor function and speech development, recurrent infections, immunodeficiency, skeletal abnormalities, destructive polyarthropathy, muscular weakness, ataxia, and psychiatric disease [1]. Alpha-mannosidosis presents in a continuum of clinical symptoms and is a heterogeneous disorder due to the varied manifestations and their different severity, as well as various rates of progression in individual patients [5]. In the majority of patients, the disease is clinically recognized in the first decade of life, progression is slow, and ataxia develops between the ages of 20–30 years [1].

Long-term enzyme replacement therapy (ERT) is a therapeutic option for alpha-mannosidosis [6] and velmanase alfa is the first human recombinant form of alpha-mannosidase available for ERT. To date, velmanase alfa (Lamzede^®^, ChiesiFarmaceutici S.p.A., Parma, Italy) is the only authorized treatment for alpha-mannosidosis in Europe; it is approved for the treatment of non-neurologic manifestations in patients with mild to moderate alpha-mannosidosis [7]. Velmanase alfa targets the underlying cause of the disease by breaking down mannose-rich oligosaccharides that would otherwise accumulate in the lysosome [8]. The velmanase alfa molecule has an identical structure to naturally occurring alpha-mannosidase and is approved for use at 1 mg/kg body weight once weekly as an intravenous infusion [7]. After infusion, velmanase alfa is taken up by cells and transported to the lysosomes, where it replaces the patient’s defective enzyme and thus reduces levels of accumulated oligosaccharides [8]. Velmanase alfa also has Orphan Drug designation in the United States and Europe.

In clinical trials, patients with alpha-mannosidosis treated with velmanase alfa achieved improvements in biomarkers, motor function, pulmonary function, immunologic profile, and quality of life (QoL) [4, 6, 9]. Importantly, a novel global treatment-response model composed of pharmacodynamic, functional, and QoL outcomes was applied post hoc to data from the pivotal randomized controlled trial (RCT) of velmanase alfa in patients with alpha-mannosidosis (NCT01681953) and the longer-term integrated data from all patients in the clinical development program. A global response required positive responses in at least two outcome domains of the model [4]. In the pivotal RCT, 13 of 15 patients (86.7%) treated with velmanase alfa achieved a global response compared with 3 of 10 patients (30.0%) who received placebo [4]. Similarly, in the integrated data set, 29 of 33 treated patients (87.9%) were global responders (100% of pediatric patients [*n* = 19] and 71% of adults [*n* = 10]) [4].

Patient registries are organized systems for collecting information that can provide uniform data for a population defined by particular diseases [10]. Establishing registries, such as those in the European Reference Networks for patients who have complex conditions [11] and the Unified European Registry for Inherited Metabolic Disorders [12], contributes to increased opportunities for coordinating regional healthcare expertise to improve the knowledge of natural disease history, and also to evaluate efficacy and safety over the long term in patients with a new treatment. SPARKLE is an alpha-mannosidosis disease registry study intended to obtain long-term safety and effectiveness data on the use of velmanase alfa during routine clinical care in patients with alpha-mannosidosis and is a post-approval commitment to marketing authorization in Europe.

## Methods

### Study design

**Fig. 1:**
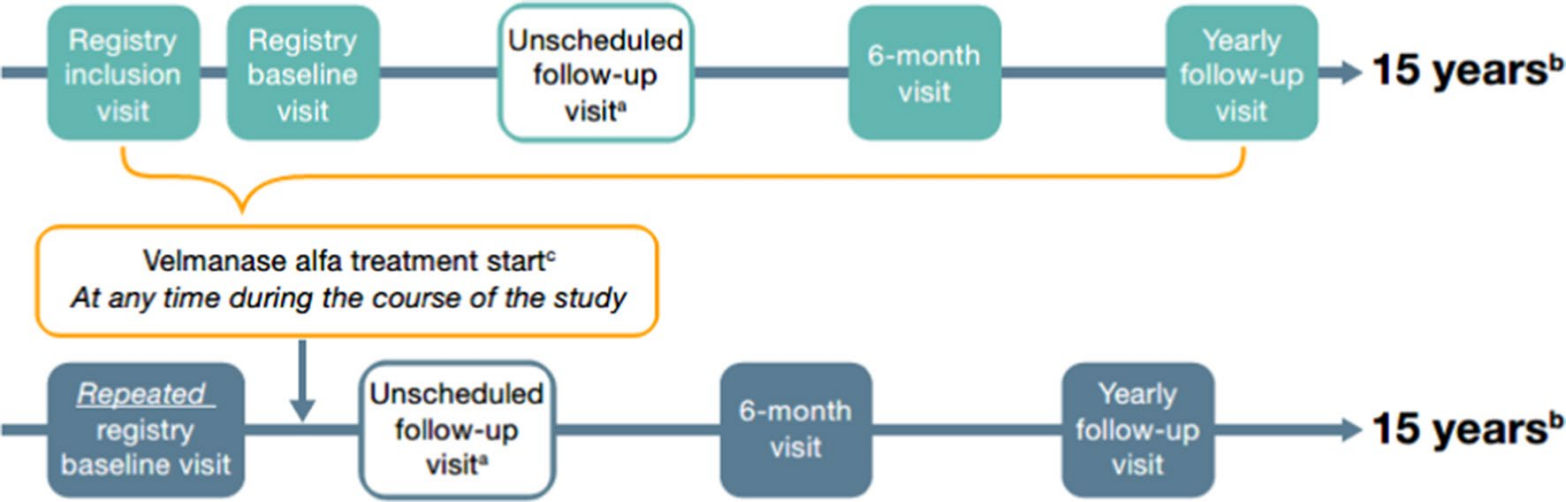
Study design. *VA* velmanase alfa. ^a^If applicable in accordance with routine clinical practice, unscheduled follow-up visits can be performed at, but not limited to, 3 months after VA treatment start, or whenever deemed appropriate according to clinician judgment for patients who start VA treatment within 1 year prior to registry inclusion. ^b^If a patient is transferred to another continuing care site during the 15-year observational period, the new treating site will be formally involved in the registry following a request of authorization submitted to the relevant ethics committee and regulatory authority to complete the 15-year observational period. ^c^Patients can start VA treatment at any time during the course of their participation in the registry; if applicable in accordance with routine clinical practice, the baseline visit should be repeated before administration of VA and unscheduled visits recommended as reported in the schema above

SPARKLE is a post-authorization safety and efficacy registry in Europe, designed as a multicenter, international, prospective cohort study of patients with alpha-mannosidosis (Fig. 1). The primary objective is to assess the long-term effectiveness and safety of treatment with velmanase alfa under conditions of routine clinical care; secondary objectives include expanding the current understanding of alpha-mannosidosis by collecting data to characterize the natural history of the disease. Source data will be collected by the investigator in the patient file and captured in an electronic case report form (eCRF). This also includes data from patients receiving treatment in a home-care setting. The study, which started patient enrollment in 2020, has an indefinite recruitment period. Patients will be followed for up to 15 years after inclusion. The end of the study is defined as the last visit of the last patient registered in the study. The study will be conducted under conditions of routine clinical practice, according to the treating physician, without mandatory registry assessments. Patients are eligible for the study irrespective of treatment (velmanase alfa, hematopoietic stem cell transplantation or any biologic substance received as part of a clinical trial during participation in the registry).

The study will be conducted in accordance with the Declaration of Helsinki, International Council for Harmonisation Good Clinical Practice, Good Pharmacovigilance Practice, and all other applicable laws and regulations of each country in which the research is carried out. The study will also be conducted in accordance with the Strengthening the Reporting of Observational Studies in Epidemiology (STROBE) guidelines. Results of the study will be reported in accordance with the Methodological Standards in Pharmacoepidemiology of the European Network of Centres for Pharmacoepidemiology and Pharmacovigilance (ENCePP) [13]. This is an observational non-interventional study capturing only data from routine clinical practice. Participation in the study will not change the patient–physician relationship, nor influence the physician’s drug prescription or therapeutic or other management of the patient.

### Patient population

Any patient in the European Union with a genetic confirmed diagnosis of alpha-mannosidosis who is willing to participate will be eligible for inclusion. Inclusion criteria are limited to a signed and dated informed consent form, including agreement to permit the Investigator to enter assessment data in the registry that has been recorded prior to inclusion in the registry, which has been obtained from the patient’s medical record. The inclusion form is signed by the patient, parents or a legally acceptable representative according to local regulation. There are no exclusion criteria for patients with this disease. Alpha-mannosidosis and its treatment will progress over the duration of the study and the study protocol may be amended as necessary. Patients’ informed consent will be required for any substantial changes.

Populations for analysis include a velmanase alfa and a no-velmanase alfa safety and efficacy analysis set. The velmanase alfa safety analysis set includes all patients in the registry who have, at some time, received any study drug treatment with a minimum individual follow-up of ≥ 1 month after the last dose. The velmanase alfa efficacy analysis set consists of patients in the same safety analysis set with efficacy evaluations during velmanase alfa treatment and for 12 months thereafter. The no-velmanase alfa safety analysis set includes patients only for the time when they are not, and have not within 1 month, been taking velmanase alfa. The no-velmanase alfa efficacy analysis set includes patients only for the time when they are not, and have not within 12 months, been taking velmanase alfa.

### Baseline and demographic variables

At the registry baseline visit (first visit after inclusion), it is suggested that the following information will be reported in the eCRF: demographics (ethnicity, age, sex), medical and disease history, genotype mutations, concomitant illnesses, previous and concomitant medications and medical procedures, physical examination (including the ability to perform the endurance test and a hearing test), vital signs, and anthropometric measurements. For patients treated with velmanase alfa, the baseline data would be taken from the time treatment was initiated in order to provide a baseline for safety and effectiveness evaluations. Therefore, it is recommended to repeat a treatment baseline visit, if in accordance with routine clinical practice, in patients starting velmanase alfa treatment at any point during participation in the registry.

### Safety variables

The primary safety outcomes of interest are rates of adverse events (AEs) in treated patients, with identified risks being anti-velmanase alfa-immunoglobulin G antibody (anti-drug antibody; ADA), infusion-related reactions, and hypersensitivity (Table 1). In all patients, treated and untreated, secondary safety outcomes and other data related to disease progression or AEs related to other drugs will be included: the evaluation of medical events (including but not limited to acute renal failure and loss of consciousness), change in vital signs (including systolic and diastolic blood pressure), laboratory tests (hematology and chemistry), physical examination, and electrocardiogram results. The suggested schedule of assessments is shown in Table 2, but assessments will be made based on the participating centers’ Standard Operating Procedures.

**Table 1:** Effectiveness and safety outcomes

Effectiveness outcomes	Safety outcomes
Laboratory assessments: • Serum oligosaccharides (µmol/L) • Serum IgG, IgA, and IgM Functional assessments: • 3MSCT (steps/min) • 6MWT (m)^a^ • 2MWT (m)^a^ • FVC (L and % of predicted)^a^ Health Assessment Questionnaires: • EQ-5D-5L • Zarit Burden Interview • QoL questionnaire • Behavior checklists for child and adult Other: • Psychotic events (rate)	• AEs: serious and nonserious • ADRs: including serious and nonserious • AEs leading to treatment discontinuation and death • Any identified risks including anti-VA-IgG antibody, infusion-related reactions, and hypersensitivity • Any potential risk of acute renal failure, loss of consciousness, and medication errors • Vital signs: SBP, DBP, and pulse rate • Electrocardiogram • Laboratory tests (hematology and chemistry) • Physical examination

*2MWT* 2-min walk test, *3MSCT* 3-min stair-climb test, *6MWT* 6-minwalk test, *ADR* adverse drug reaction, *AE* adverse event, *DBP* diastolic blood pressure, *FVC* forced vital capacity, *FVC%* forced vital capacity, percent of predicted, *Ig* immunoglobulin, *QoL* quality of life, *SBP* systolic blood pressure, *VA* velmanase alfa

^a^In patients < 4 years of age, 3MSCT, 2MWT, and FVC% of predicted will be proposed based on physician judgment

**Table 2:** Suggested schedule

Procedure	Registry inclusion visit (~ –7 days)	Registry baseline visit (time 0)	Unscheduled follow-up routine clinical visit^a^	Six-month follow-up routine clinical visits^b^	Yearly follow-up routine clinical visits
*Administrative*					
Informed consent form	X	X^c^			
Inclusion/exclusion criteria	X	X^c^			
Medical and disease history (including disease onset, residual enzymatic activity, genotype mutations, bone marrow transplantation) and concomitant illnesses	X	X^c,d^			
Previous and concomitant medications, including VA in hospital or home infusion setting		X^d^	X	X	X
Concomitant procedures	X	X	X	X	X
Physical examination^e^ (vital signs and anthropometric measurements, including rate of growth)		X	X	X	X
Demographics	X	X^c^			
*Administration of medication*					
VA therapy, in hospital or home infusion setting^f^		X	X	X	X
*Assessments*^g^					
Standard hematological tests^h^		X	X		X
Laboratory chemistry^i^		X	X		X
Anti-VA-IgG antibody (ADA)^j^		X	X		X
Oligosaccharides in serum^j^		X	X		X
Serum IgG, IgA, IgM^j^		X	X		X
3MSCT, when applicable^k^		X			X
6MWT, when applicable^k^		X			X
2MWT, when applicable^l^		X			X
FVC (L and % of predicted)		X	X		X
Electrocardiogram		X	X		X
Hearing test (PTA)		X	X		
Psychotic events		X	X		X
AEs/ADRs^m^	X	X	X	X	X
*Health Assessment Questionnaires*					
EQ-5D-5L		X			X
Zarit Burden Interview		X			X
CHAQ		X			X
Behavior checklists^n^		X			X

*3MSCT*3-min stair climbing test, *2MWT*2-min walk test, *6MWT*6-min walk test, *ABCL* Adult Behavior Checklist, *AE* adverse event, *ADA* alfa-immunoglobulin G antibody, *ADR* adverse drug reaction, *ALP* alkaline phosphatase, *ALT* alanine aminotransferase, *ASR* Adult Self-Report, *AST* aspartate transaminase, *CBCL* Child Behavior Checklist, *CHAQ* Childhood Health Assessment Questionnaire, *FVC* forced vital capacity, *Ig* immunoglobulin, *L* liter, *LDH* lactate dehydrogenase, *OABCL* Older Adult Behavior Checklist, *PTA* pure tone audiometry, *RMP* risk management plan, *VA* velmanase alfa

Actual assessments performed per participating center Standard Operating Procedures

^a^Unscheduled follow-up visits can be performed at, but not limited to, 3 months after VA treatment start, or whenever deemed appropriate according to treating physician’s judgment for patients who start VA treatment within 1 year prior to Registry inclusion

^b^Additional follow-up visit after 6 months is highly recommended for patients who start VA treatment within 1 year prior to registry inclusion

^c^If not taken or collected at registry inclusion visit

^d^Patients’ retrospective data may be collected at the time of registry enrolment if available. Baseline visits are recommended to be repeated for those patients who will start VA therapy during the course of the study, before therapy is initiated

^e^Physical examination to collect vital signs, anthropometric measurements like height, weight, rate of growth, and the ability to perform the endurance test at the study visit

^f^If applicable, information regarding weekly VA therapy received by the patient need to be recorded within all the registry duration

^g^All assessments mentioned in the above table will be collected only if considered necessary and available as part of routine clinical practice by the treating physician; no additional tests specific to the registry will be done

^h^Standard test for hematology assessed by local laboratory if available per clinical practice of the site: hemoglobin, hematocrit, platelet count, red blood cells, and white blood cells with differential count (all expressed in %, as well as in absolute numbers)

^i^Standard test for hematology assessed by local laboratory if available per clinical practice: serum electrolytes, creatinine, creatine-kinase, amylase, AST, ALT, ALP, albumin, bilirubin (total and direct), LDH

^j^The markers (oligosaccharides in serum, IgG, IgA, and ADA) will be evaluated through a central laboratory in European countries other than Germany. In Germany, marker testing will take place according to local routine clinical practice

^k^In patients 4 years and older, and when applicable according to the judgment of treating physician

^l^In patients under 4 years of age and when applicable according to the judgment of treating physician

^m^AEs/ADRs based on all risk categories associated with VA European RMP, including infusion-related reactions and hypersensitivity (as identified risks), acute renal failure, loss of consciousness and medication errors (as potential risks). Data on pregnancy (including birth and newborn data) and lactation (as missing information) will be also collected if available

^n^One of the behavior checklists (CBCL pre-scholar/scholar, ABCL/ASR [self-reporting], OABCL) will be used to the applicable age and according to the judgment of treating physician

### Effectiveness variables

Efficacy outcomes to be assessed during routine clinical care are shown in Table 1. For patients under 4 years of age, a 3-min stair climb test (3MSCT) and 2-min walk test (2MWT) are suggested. The forced vital capacity (% volume predicted) is suggested for patients over 4 years of age, and according to the judgment of the treating physician. The proposed schedule of assessments is shown in Table 2, but, again, assessments will be made based on the participating centers’ Standard Operating Procedures.

A global treatment response (GTR) to velmanase alfa will be evaluated in velmanase alfa-naïve patients able to perform the functional tests after 3 years of treatment. The GTR will assess patients in three clinical domains (pharmacodynamic, functional, and QoL) consisting of 1 or > 1 endpoint (Table 3). The pharmacodynamic domain comprises a measure of the accumulated substrate in alpha-mannosidase deficiency, the main pathogenic factor for disease manifestations; a decrease in oligosaccharides is a key biomarker of the pharmacologic effect of the ERT [4]. The second domain includes pulmonary function, endurance, and fine and gross motor proficiency, i.e., all the functional endpoints of the disease [4]. The QoL domain relates to patient-related outcomes of disease burden, disability, and pain [4]. A global response is defined as improvements exceeding the established minimal clinical important difference (MCID) (Table 3) in ≥ 1 endpoint in ≥ 2 of the domains [4]. The MCIDs are derived on the basis of those used in clinical conditions with the highest similarity to alpha-mannosidosis (proxy diseases) [14–17]. Collection of the same effectiveness variables is suggested for patients who are not treated with velmanase alfa, in order to support the description of the natural course of the disease.

**Table 3:** Clinical domains, endpoints, and MCID thresholds for the GTR

Domain	Endpoint	Established MCID^a^
Pharmacodynamic	Serum oligosaccharides	Last serum oligosaccharide value ≤ 4 µmol/L
Functional	3MSCT	Improvement ≥ 7 steps/min [14]
	6MWT	Improvement ≥ 30 m [16]
	FVC%	Improvement ≥ 10% [15]
Quality of life	CHAQ DI	Improvement ≤ –0.130 [17]
	CHAQ Pain VAS	Improvement ≤ –0.246 [17]

*3MSCT* 3-min stair-climb test, *6MWT* 6-min walk test, *CHAQ DI* Child Health Assessment Questionnaire Disability Index, *CHAQ Pain VAS* Child Health Assessment Questionnaire Pain Visual Analog Scale, *FVC%* forced volume capacity, percentage of predicted, *GTR* Global Treatment Response, *MCID* minimal clinically important difference, *MPS IV A* mucopolysaccharidosis type IV A

^a^MCID were derived from proxy diseases (3MSCT from MPS IV A clinical trials; 6MWT and FVC% from Pompe; CHAQ DI and CHAQ Pain VAS from juvenile arthritis)

### Data management

Data sources will be the patient’s medical record and any other medical record of clinical findings accrued as part of routine care; retrospective data may be obtained from the patient’s medical record. All data related to the study will be entered in the eCRF, housed on IBM Clinical Development, and will be checked for accuracy and completeness as a component of ongoing study monitoring by an independent contract research organization (CRO) designated by Chiesi Farmaceutici S.p.A. Access to the eCRF will be granted to authorized personnel; sites will only be able to access data of patients enrolled at their site and the CRO will be entitled to access all data in a read-only mode.

For patients who discontinue the study, all efforts will be made to complete and report the observations as thoroughly as possible. If a patient is transferred to another continuing care site, the new treating site will be formally involved in the study after a request of authorization submitted to the relevant ethics committee and regulatory authority. In case of withdrawal, the investigator must report the main reason for withdrawal.

### Study size

As this is an observational study and there is no formal hypothesis testing, the size of the study was conceptualized based on assumed different proportions of GTR in treatment-naïve patients who can perform functional tests after 3 years of treatment. Assuming at least 70 treated patients are enrolled, and assuming a GTR of at least 0.60, the lower limit of the two-sided 95% confidence interval (Clopper–Pearson) is always above 45.0% (Table 4), which is considered a clinically relevant response. Thus, the efficacy analysis is based on a hypothetical treated population of at least 70 patients.

**Table 4:** Efficacy analysis based on hypothetical treated population of at least 70 patients

Expected proportion	95% CI lower limit	95% CI upper limit	95% actual width
0.60	0.476	0.715	0.239
0.65	0.527	0.760	0.234
0.70	0.579	0.804	0.225
0.75	0.632	0.846	0.214
0.80	0.687	0.886	0.199

*CI* confidence interval

### Statistical analyses

Baseline demographic and disease characteristics will be presented by means of descriptive statistics and frequency distributions, as appropriate. Descriptive safety outcomes will be presented and summarized over time. For AEs, both the frequency of patients with an AE and the number of events will be presented by year from baseline. Safety variables will be presented based on person-years at risk. Effectiveness variables will be summarized by means of descriptive statistics or frequency of distribution, as appropriate, and all variables (actual values and change from baseline, if applicable) will be presented by time point. The effects of baseline characteristics on effectiveness will be evaluated by means of regression/logistic models. Covariates in the model will be age (< 18 and ≥ 18 years), sex (male, female), sibships, genotype (Groups 1, 2, and 3, per Borgwardt et al.) [18], residual enzymatic activity (< 10, 10 to < 15, and ≥ 15 nmol/h/mg), and Childhood Health Assessment Questionnaire Disability Index (0–1, 1–2, and 2–3).

Provided that a sufficient number of nontreated patients with adequate data are enrolled, a comparison will be conducted between the velmanase alfa-treated and nontreated groups. Propensity score (PS) analysis will be used to compare GTRs after 3 years; the PS will be calculated as the predicted probability of response using logistic regression, with the same covariates as used for the baseline characteristics regression. Subgroup analyses may be performed depending on overall sample size; results will be presented descriptively.

## Discussion

SPARKLE is the first European-wide registry for alpha-mannosidosis that includes all patients, irrespective of treatment or study inclusion, who are willing to participate. Currently, limited information is available on the natural history of alpha-mannosidosis, with only one study published to date [4]. SPARKLE allows the possibility of monitoring patients over 15 years, with the objective of broadening the current understanding of alpha-mannosidosis heterogeneity, disease progression, burden and—potentially—life expectancy. The registry will expand the current understanding of alpha-mannosidosis by collecting natural history data, disease modifiers, and confounding factors in treated and untreated patients. Rare disease registries provide a valuable resource to enhance understanding of the diagnosis, heterogeneity, progression, burden of disease, and natural history of these conditions [19]. In addition, they help to characterize and describe patients, evaluate the long-term safety and effectiveness of available treatment options, and support measures to optimize patient care through recommendations for monitoring and development of treatment strategies. SPARKLE will support monitoring of patients treated with velmanase alfa in routine clinical care, further assessing its safety and effectiveness.

SPARKLE is part of a growing number of rare disease registries, with over 750 listed in Europe as of May 2019 [20]. SPARKLE will contribute to the increasing database of information gathered from lysosomal storage disease registries, including those for Fabry disease, Pompe disease, Gaucher disease, and mucopolysaccharidoses [21, 22], as well as the almost 40 registries/databases that may include patients with alpha-mannosidosis registered with Orphanet [21]. Importantly, SPARKLE is the only ongoing registry study specifically designed for alpha-mannosidosis; it will provide a much-needed data set relating to this disease, and benefits from several strengths. The protocol adheres to the rare disease registry guidelines suggested by the International Rare Diseases Research Consortium [23] and recommendations of the European Union Committee of Experts on Rare Diseases [24]. Existing treatment registries that evaluate orphan medicinal products for the treatment of lysosomal storage disorders are often limited by incompleteness and variable quality of the data [25]. The eligibility criteria for participation in SPARKLE are minimal in order to allow the largest possible number of patients to participate, and methodologic rigor will be exercised to ensure the highest-quality data and data analysis. In addition, SPARKLE is the only European alpha-mannosidosis-specific registry; it is designed to collect both safety and efficacy data in treated patients, as well as information on patient and disease characteristics. It is not restricted to patients receiving a particular treatment, or a time when treatment is initiated.

Some limitations need to be acknowledged. As information in the registry will be collected during routine clinical care, the assessments performed and data collected may vary from center to center, as may the interpretation of data by the individual Investigators. In addition, potential bias of unknown size and direction could be introduced by patient self-selection into the study and by the fact that some patients with alpha-mannosidosis may be treated in centers not participating in the registry.

## Conclusion

The SPARKLE study will provide real-world data in European patients with alpha-mannosidosis during routine clinical care and will increase the understanding of the natural course, clinical manifestations, and progression of this ultra-rare disease in both treated and untreated patients and specifically on the long-term safety and effectiveness of velmanase alfa treatment.

## Data Availability

Not applicable.

## Abbreviations

2MWT: 2-min walk test
3MSCT: 3-min stair climbing test
6MWT: 6-min walk test
ABCL: Adult Behavior Checklist
ADA: Alfa-immunoglobulin G antibody
ADR: Adverse drug reaction
AE: Adverse event
ALP: Alkaline phosphatase
ALT: Alanine aminotransferase
ASR: Adult Self-Report
AST: Aspartate transaminase
CBCL: Child Behavior Checklist
CHAQ: Childhood Health Assessment Questionnaire
CHAQ: DI Child Health Assessment Questionnaire Disability Index
CHAQ Pain VAS: Child Health Assessment Questionnaire Pain Visual Analog Scale
CI: Confidence interval
CRO: Clinical research organization
DBP: Diastolic blood pressure
eCRF: Electronic case report form
ERT: Enzyme replacement therapy
FVC: Forced vital capacity
FVC%: Forced vital capacity percent of predicted
GTR: Global treatment response
Ig: Immunoglobulin
L: Liter
LDH: Lactate dehydrogenase
MCID: Minimal clinically important difference
MPS IV A: Mucopolysaccharidosis type IV A
OABCL: Older Adult Behavior Checklist
PS: Propensity score
PTA: Pure tone audiometry
QoL: Quality of life
RCT: Randomized controlled trial
RMP: Risk management plan
SBP: Systolic blood pressure
VA: Velmanase alfa
